# Sterically Hindered Quaternary Phosphonium Salts (QPSs): Antimicrobial Activity and Hemolytic and Cytotoxic Properties

**DOI:** 10.3390/ijms23010086

**Published:** 2021-12-22

**Authors:** Vadim V. Ermolaev, Daria M. Arkhipova, Vasili A. Miluykov, Anna P. Lyubina, Syumbelya K. Amerhanova, Natalia V. Kulik, Alexandra D. Voloshina, Valentine P. Ananikov

**Affiliations:** 1 Arbuzov Institute of Organic and Physical Chemistry, FRC Kazan Scientific Center, Russian Academy of Sciences, Arbuzov str., 8, Kazan 420088, Russia; ermolaev@iopc.ru (V.V.E.); miluykov@iopc.ru (V.A.M.); anna.lyubina@iopc.ru (A.P.L.); syumbelya.gumerova@iopc.ru (S.K.A.); natylick2017@mail.ru (N.V.K.); sobaka-1968@mail.ru (A.D.V.); 2 N.D. Zelinsky Institute of Organic Chemistry, Russian Academy of Sciences, Leninsky Prospect, 47, Moscow 119991, Russia; val@ioc.ac.ru

**Keywords:** phosphonium ionic liquids, QPS, antimicrobial activity, fungicidal activity, cytotoxicity, hemolytic properties

## Abstract

Structure–activity relationships are important for the design of biocides and sanitizers. During the spread of resistant strains of pathogenic microbes, insights into the correlation between structure and activity become especially significant. The most commonly used biocides are nitrogen-containing compounds; the phosphorus-containing ones have been studied to a lesser extent. In the present study, a broad range of sterically hindered quaternary phosphonium salts (QPSs) based on tri-*tert*-butylphosphine was tested for their activity against Gram-positive (*Staphylococcus aureus, Bacillus cereus*, *Enterococcus faecalis*) and Gram-negative (*Escherichia coli*, *Pseudomonas aeruginosa*) bacteria and fungi (*Candida albicans*, *Trichophyton mentagrophytes* var. gypseum). The cation structure was confirmed to determine their biological activity. A number of QPSs not only exhibit high activity against both Gram-positive and -negative bacteria but also possess antifungal properties. Additionally, the hemolytic and cytotoxic properties of QPSs were determined using blood and a normal liver cell line, respectively. The results show that tri-*tert*-butyl(*n*-dodecyl)phosphonium and tri-*tert*-butyl(*n*-tridecyl)phosphonium bromides exhibit both low cytotoxicity against normal human cells and high antimicrobial activity against bacteria, including methicillin-resistant strains *S. aureus* (*MRSA*). The mechanism of QPS action on microbes is discussed. Due to their high selectivity for pathogens, sterically hindered QPSs could serve as effective tunable biocides.

## 1. Introduction

The widespread use of antibiotics for the treatment of various diseases of nonbacterial etiology and other forms of misuse have led to the appearance of bacterial pathogens that are resistant to many well-known antibiotics [1,2,3,4]. The exceptional evolution of ESKAPE pathogens (*Enterococcus faecium*, *Staphylococcus aureus*, *Klebsiella pneumoniae*, *Acinetobacter baumannii*, *Pseudomonas aeruginosa*, and *Enterobacter* species) has led to the emergence of “superbugs”, which have gained an extraordinary level of resistance [5,6]. The application of antibiotics has also led to the destruction of the human biota, resulting in reduced immune system activity and facilitating infection [7]. Annually, around a quarter of all registered deaths across the world are caused by viruses, bacteria and fungi [8,9]. The above-mentioned problems necessitate a rational approach to the design of a new generation of antibiotics [10].

Many distinct biocidal agents are known [11]. Among the most promising and widely studied are cationic surfactants, namely quaternary heteronium salts [12,13,14]. It is well known that their antimicrobial activity depends on the density of the cationic charge and the hydrophobicity of the active molecule. In the case of cationic surfactants, the determinative factors are the charge, the number of cationic centers, the nature of the counter-ion, and the length/number of alkyl substituents and aromatic groups [15]. These structure features regulate the self-organization of the surfactants, increasing the local concentrations and cationic charges of amphiphiles on bacteria. The so-called “cut-off effect” in a homologous series of surfactants with long hydrocarbon chains should be noted: as the chain length increases, the biological activity of the compounds increases up to a critical point, beyond which the activity is lost [16].

An equally important aspect of the biological properties of new compounds is cytotoxicity [17]. The balance between strong antimicrobial properties and low toxicity to normal cells is the basis of the search for new effective antimicrobial agents. Compounds with strong antimicrobial effects cannot be approved if they exhibit high cytotoxicity to healthy eukaryotic cells.

Among the most prevalent bactericides are various quaternary ammonium salts (QASs) [18]. They are also active against bacteria, fungi, parasites and even lipophilic viruses [19]. Generally, they disrupt the cell membranes of micro-organisms [20,21]. The long lipophilic substituent and the charged center of QAS allow it to become incorporated into the phospholipid bilayer, destroying the cell membrane and acting on bacteria regardless of the species. Therefore, QASs are active against multidrug-resistant strains such as *Enterococcus faecium*, *Staphylococcus aureus*, *Klebsiella pneumoniae*, *Acinetobacter baumannii* and *Pseudomonas aeruginosa* [6]. Some additional possible mechanisms of the influence of biocides on the pathogenic cells are proposed [22].

Quaternary phosphonium salts (QPSs) are similar in their structure and biological activity against microbes [23,24]. Their bactericidal activity and safety for humans were discovered in the middle of the 20th century [25] and continue to be studied [26]. Particularly intensive research on their biological activity has been spurred by the spread of the usage of phosphonium ionic liquids (PILs) in science and technology. Seddon et al. studied the antimicrobial activity of the series of tri-*n*-hexyl(R)phosphonium salts with even numbers of carbon atoms (from C2 to C16) in the fourth alkyl substituent of normal structure [27]. It was found that the alkyl substituent length was responsible for the antimicrobial properties of PILs. The effect of the nature of the anion on the antimicrobial activity of the phosphonium compound was investigated in the example of tri-*n*-hexyl(*n*- tetradecyl)phosphonium salts. The results were compared to those of benzalkonium chloride for a broad spectrum of micro-organisms: cocci, bacilli, rods and fungi.

Another study showed that tri-*n*-hexyl(*n*-tetradecyl)phosphonium salts that were coupled with various anions met all the requirements of new compounds for pharmaceutical use. Their antibacterial, anticancer and antioxidant activities were also demonstrated [28].

Phosphonium salts based on triphenylphosphine have been studied to a greater extent. Apart from a broad spectrum of biological activity, they exhibit strong antifouling properties [29]. Various triphenyl(R)phosphonium compounds have demonstrated biological activity: naphtoquinone derivatives [30], carboxylated betaines with an alkyl substituent [31] and metronidazole derivatives [32]. One of the areas of research into the biological activity of QPSs is the study of dicationic compounds based on bis(triphenylphosphonium) salts [17,33]. Zhang et al. showed the higher antibacterial activity of dicationic compared to monocationic salts.

It should be mentioned that phosphonium compounds have also been applied as bactericides that are covalently bonded to plastic surfaces or used as co-extrusive additives for plastic materials in goods production [34]. Such surfaces are confirmed to demonstrate antibacterial properties. Another work studied a plastic surface with a bonded phosphonium salt containing an oxanorbornene moiety [35]. Of the various compounds, tri-*p*-methoxyphenylphosphine was the most active against bacteria, but it also demonstrated the highest hemotoxicity.

Quaternary phosphonium salts act as potential antimicrobial agents with a number of notable benefits [36]. However, the systematic study of the structure–property relationships and the development of a strategy for the regulation of biological properties through the structural tuning of active molecules is still required. Notably, the key to the optimization of potential antimicrobial agents remains the balance between antimicrobic activity and cytotoxicity. The peculiar structure of the sterically hindered salt cation, its charge distribution and its shielding of charged phosphorus atoms by *tert*-butyl groups differ greatly from the classical quaternary heteronium salts, allow the hypothesis of distinctive biological properties and different mechanisms of interaction with living cells [37]. Nowadays, a few sterically hindered QPSs are known [38,39] and their biological activity is not studied.

Previously, our group synthesized a series of QPSs based on tri-*tert*-butylphosphine for the first time, which were characterized using a full spectrum of physical–chemical methods [40,41,42]. A direction of research that follows naturally from that study is to investigate the biological activity of those compounds. In the present study, we characterized the antimicrobial, hemolytic and cytotoxic activity of the sterically hindered QPSs and determined the relationship between the structure and biological activity of these QPSs for the first time. We tested the use of the lead compound as an antimicrobial coating on test plastic products that were fabricated using fused-deposition-modeling (FDM) technology.

## 2. Results and Discussion

### 2.1. Antimicrobial and Fungicidal Activity

Tri-*tert*-butyl(*n*-alkyl)phosphonium salts contain a sterically hindered head group with a cationic charge on the phosphorus atom and a linear aliphatic “tail” that imparts hydrophobic properties to the compounds. Halide ions and a weakly coordinating tetrafluoroborate anion were used as counter-ions. The structures and designations of the QPSs are summarized in Table 1. The first members of the QPSs **1a,b–3a,b** are high-melting-point white powders; most have melting points around 100–120 °C. It should be noted that the salts **11a–17a** are ‘ionic liquids’ in terms of the classical definition and melt below 100 °C. With an increase in the length of the alkyl substituent, the hydrophobicity of the molecules increases. QPSs with halide anions are highly soluble in water, although their solubility decreases with an increase in molecular weight. Conversely, the phosphonium tetrafluoroborates are almost insoluble in water, with the first five members being weakly soluble exceptions (**1b–5b**). To increase their solubility, a 5% aqueous solution of DMSO was used.

The synthesized compounds were tested for antimicrobial activity against a number of Gram-positive bacteria, *Staphylococcus aureus* (*Sa*), *Bacillus cereus* (*Bc*) and *Enterococcus faecalis* (*Ef*), and Gram-negative bacteria, *Escherichia coli* (*Ec*) and *Pseudomonas aeruginosa* (*Pa*), including the methicillin-resistant strains *S. aureus MRSA-1* (resistant to fluoroquinolones and beta-lactams) and *MRSA-2* (resistant to beta-lactams). The methicillin-resistant strains *S. aureus* were transferred by the Republican Clinical Hospital of the Republic of Tatarstan (Kazan). The fungicidal activity against *Candida albicans* (*Ca*) and *Trichophyton mentagrophytes* var. gypseum (*Tm*) was studied. These pathogens were chosen because they are the most widespread in the food industry and the healthcare system.

The results are shown in Table 2 (and Table S1 in the Supplementary Materials for QPSs with 1–9 carbon atoms in the alkyl substituent). The compounds **1a,b–5a,b** do not possess antimicrobial activity; the minimum inhibitory concentrations (MICs) of these compounds exceed 500 μg/mL, which is quite likely due to the short length of their alkyl substituents and their consequent low lipophilicity, rendering them ineffective. With an increase in the lipophilicity of the alkyl substituent, the ionic compounds **6a,b–18a,b** and **20a,b** start to show antimicrobial activity that reaches a maximum for **11a,b–18a,b**. The MICs of the last compounds against Gram-positive bacteria (*S. aureus*, *B. cereus* and *E. faecalis)* were in the range of 0.12–0.98 μg/mL, which is at the level of the widely used antibiotic of the fluoroquinolone series, ciprofloxacin. It should be noted that these QPSs also demonstrated high antimicrobial activity against *MRSA* strains.

Compounds **14a,b–16a,b** were the most active against the Gram-negative bacteria *Escherichia coli* and *Pseudomonas aeruginosa*. The antifungal activity was most pronounced for compounds **15a,b–18a,b** and manifested at the level of the reference drug ketoconazole. Interestingly, most of the leading compounds acted both bactericidally and fungicidally; their MICs and MBCs (MFCs) differed from each other by no more than four times. The difference in antimicrobial activity between halide and tetrafluoroborate anions was minor, affirming the determining role of the cationic nature in the biological activity of this comparison series.

It should be mentioned that the “cut-off effect”—whereby, with an increase in the number of methylene fragments in the alkyl substituent, the compounds increase in biological activity up to a critical point, beyond which they cease to be effective—was not observed in the range of sterically hindered phosphonium salts that was studied. The biocidal activity of QPSs with alkyl chain lengths of 18 and 20 carbon atoms was lower, but these compounds were still active against bacteria and fungi.

### 2.2. Hemolytic and Cytotoxic Activity of QPSs

**Figure 1:**
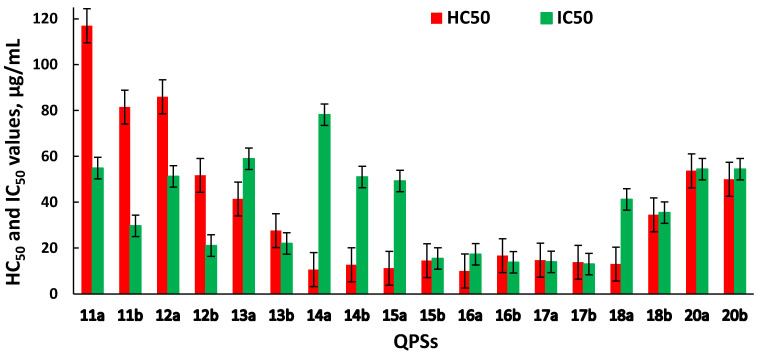
Hemotoxic and cytotoxic activity of QPSs, expressed in terms of HC_50_ and IC_50_.

An important characteristic when assessing the biological activity of new chemical compounds is their cytotoxic effect on eukaryotic cells. The ability of an investigated compound to cause the destruction of human erythrocytes illustrates its toxic effect on the internal environment. The hemolysis assay is a simple screening test that can help in the study of cytotoxicity in more complex models. Cell lines obtained from various human organs and tissues that allow for adequate estimation of the effects of new potential drugs on cell metabolism can serve as experimental models. Hence, the sterically hindered QPSs were tested for cytotoxicity against red blood cells and the human hepatocyte Chang liver cell line (Figure 1).

Data on hemolytic and cytotoxic activity are represented by the HC_50_ (the concentration of the test compound that causes the 50% hemolysis of erythrocytes in the experiment) and IC_50_ (the concentration of the test compound that causes the death of 50% of the cells in the test population) values, respectively.

Compounds **1a,b–10a,b**, across the tested range of concentrations, did not exhibit high hemolytic or cytotoxic activity, with HC_50_ > 125 µg/mL and IC_50_ ranging from 66 to >100 µg/mL (Table S2 in Supplementary Materials).

It was found that the manifestation of hemolytic activity by the tested compounds depended on the cation structure, while the anion nature did not dramatically influence the hemotoxicity (Figure 1). The compounds **11a,b–13a,b** exhibited weaker hemotoxic properties, while their antibacterial activity was still very high. With an increase in the lipophilicity of the alkyl substituents (**14a,b–17a,b**), the hemolytic effect slightly decreased. However, at the same time, there was an increase in antibacterial and antifungal activity in a number of cases. A further increase in the alkyl substituent length (**18b, 20a,b**) led to a decrease in both hemotoxicity and antimicrobial activity. The exception was compound **18a**, for which the hemolytic properties of which did not change, and high antibacterial and antifungal activity was demonstrated.

Against normal liver cells (Chang liver), the compounds **11a–15a** were observed to be less toxic than their counterparts with tetrafluoroborate anion. Apparently, in this case, the change in cytotoxic properties was associated with the nature of the counter-ion. Meanwhile, a dependence on the anion nature for phosphonium compounds **16a,b–18a,b** and **20a,b** was not pronounced. In some cases, the HC_50_ and IC_50_ values for compounds with different counter-ions were similar; however, in other examples, they differed by about 5–7 times. Compounds **12a** and **13a** should be especially noted, as they showed the lowest cytotoxicity against erythrocytes and human liver cells and, at the same time, very high antimicrobial activity against the tested strains of micro-organisms, including *MRSA*.

**Figure 2:**
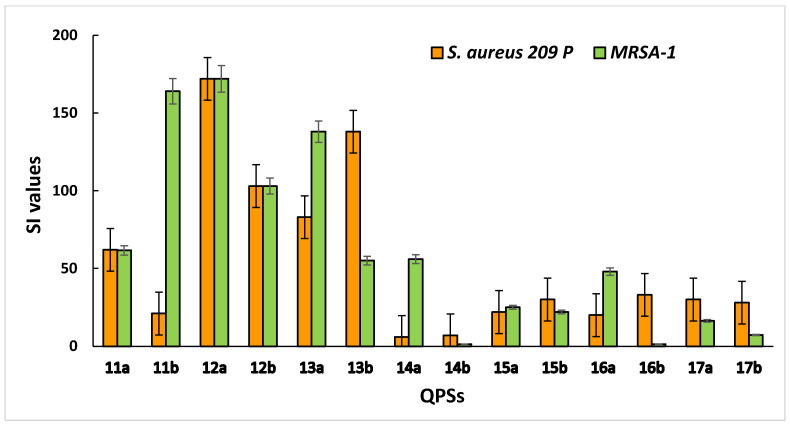
Selectivity of QPSs for bacteria (*S. aureus 209 P* and *MRSA-1*) compared to red blood cells.

The selectivity of compounds for microbial cells is an important criterion for assessing the cytotoxic effects. This indicator is characterized by the selectivity index (SI), which was calculated as the ratio between the HC_50_ value for erythrocytes (IC_50_ for eukaryotic cells) and the MBC value for bacterial cells. It was found that compounds **12a,b** and **13a,b** showed the highest selectivity for the *S. aureus 209 P* and *MRSA-1* strains, with low toxicity to human blood and liver cells (Figure 2).

**Figure 3:**
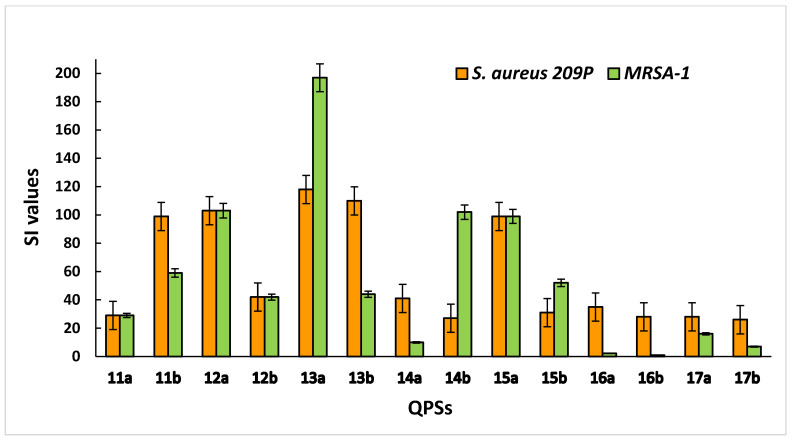
Selectivity of QPSs for bacteria (*S. aureus 209 P* and *MRSA-1*) compared to Chang liver cells.

For liver cells, the highest selectivity was shown by compounds **12a**, **13a** and **15a** (Figure 3). Overall, most of the studied QPSs showed outstanding selectivity for the *S. aureus 209 P* and *MRSA* strains while demonstrating low toxicity to human erythrocytes and hepatocytes.

The results obtained for the investigated phosphonium salts demonstrates their high antimicrobial activity and selectivity for microbes as well as their safety for normal human cells, which is a prerequisite for the development of potential new antimicrobial agents.

### 2.3. Analysis of CV Absorbance Spectra

The study of the mechanism of action is one of the most important stages in the investigation of new antimicrobial agents. According to a number of authors, the mechanisms of action of QPSs could be associated with the disruption of the structure of the bacterial cytoplasmic membrane, leading to a change in its permeability [43].

**Figure 4:**
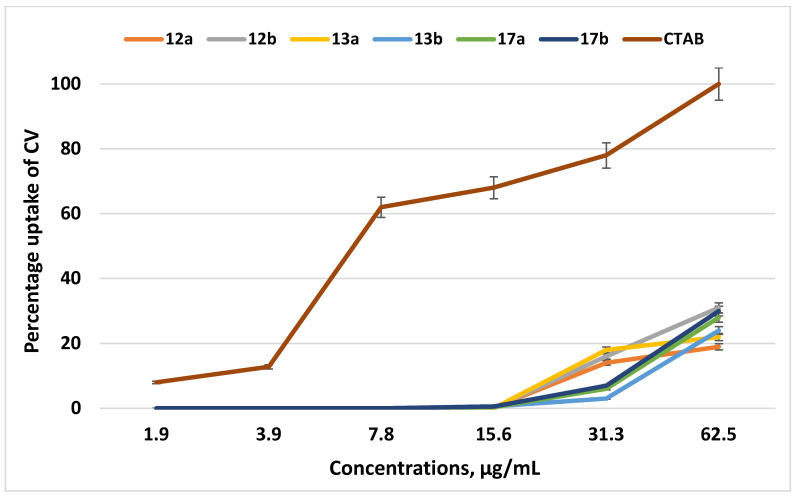
The percentage of crystal violet in *S. aureus 209 P* supernatant after 30 min incubation with various concentrations (µg/mL) of the test compounds and CTAB. The optical density of the sample at a wavelength of 540 nm with the dye in the absence of cells was taken as 100%.

Therefore, we studied the membranotropic effect of the QPSs on the absorption of the crystal violet (CV) dye, since an increase in the ability of the CV dye to penetrate implies a change in the permeability of the cytoplasmic membrane (BMC). The experiment was carried out for the examples of the lead compounds **12a,b** and **13a,b**; QPSs with longer alkyl substituents, **17a,b**, were also chosen for comparison. The traditional membranotropic agent cetyl(trimethyl)ammonium bromide (CTAB) was used as a control. Figure 4 demonstrates that all of the tested compounds in the ranges of their MICs and MBCs (1.9–62.5 μg/mL) had very poor effects on the permeability of the cytoplasmic membrane of *S. aureus 209 P*. At the highest concentrations of the lead compounds, which were many times higher than the MICs and MBCs, the uptake of CV did not exceed 30%.

It should be noted that the tested compounds, throughout the ranges of studied concentrations, did not have a significant effect on the structure of the cytoplasmic membrane of *S. aureus 209 P* compared to the classic CTAB surfactant. In this regard, one can assume that the specific mechanisms of action of the tested compounds in the concentration ranges, corresponding to the MICs and MBCs, differ from the actions of classical cationic surfactants and could be associated with their ability to disrupt the energy metabolism in bacteria due to the induction of a sharp decrease in the membrane potential by stimulating protonophoric uncoupling [44]. The difference of tri-*tert*-butyl(*n*-alkyl)phosphonium salts from classical biocides based on non-hindered heteronium salts could consist of the structure, arrangement and charge distribution of the cation.

### 2.4. Antimicrobial Coating of 3D-Printed Polymeric Surfaces

There are incredible advantages to using 3D-printing technology for creating customized products with precisely defined parameters. In many cases, biocidal properties are necessary for such products, but they are not limited to uses in medicine [45,46,47,48]. Imparting antimicrobial properties to surfaces, as well as preventing the formation of biofilms and their fouling by resident living multicellular organisms, is of great practical importance. There are several approaches to imparting bactericidal properties to surfaces: the physical modification of the surface, the use of additives in the material [49], or the attachment of biocides to the surface [50]. The most affordable, available and effective method is the surface treatment of finished products. This method, in contrast to the use of co-extrusive additives, does not change the construction properties of the materials and does not require incorporation into the production process.

There are two types of coatings. For the first, the coating is covalently bonded to the protected surface, and in the second case, the biocide-release mechanism is implemented when biocide not only acts on the surface but also creates an inhibition zone [50].

In order to study the possibility of using QPSs as surface biocides for FDM polymers, the six materials most commonly used for 3D printing (Table 3) were coated with the lead compound **12a** and subjected to testing.

The plastic samples, which were 10 mm × 10 mm × 2 mm, were preliminary sterilized and placed into an ethanolic solution of **12a** for 1 min. Then, the studied and control (without coating) samples were immersed in bacterial suspensions with a known concentration of bacteria (10^5^–10^8^ bacteria/mL) for 24 h, and the number of viable micro-organisms was then estimated [51]. The results are shown in Table 4.

The highest bactericidal effect for **12a** was achieved on the surfaces of ABS, PLA natural and PETG plastics (Table 4). At a concentration of 0.01%, a complete absence of bacterial growth was observed. Upon reducing the concentration of the compound to 0.001%, the best bactericidal effect was observed for PETG transp. The bacterial cell concentration was decreased by 27 times compared to the control.

The present experiment is the first step in developing potential QPSs for the coating of plastic products. Further investigation would include the addition of phosphonium compounds to the plastic mass to provide it with biocidal properties in order to obtain antiseptic materials.

### 2.5. Zone-of-Inhibition Test

**Figure 5:**
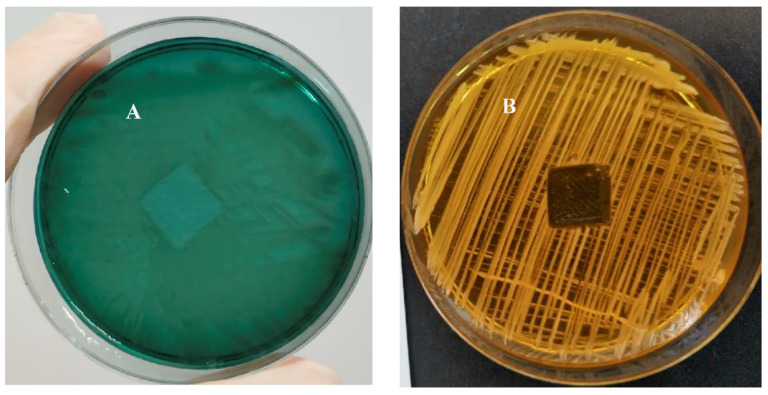
Picture of a zone-of-inhibition-test result of 1% **12a** coating plastic sample (PC+); (**A**) zone of inhibition of *P. aeruginosa* and (**B**) zone of inhibition of *S. aureus*.

The inhibition-zone test is a quick and qualitative way to measure the ability of an antimicrobial agent to suppress microbial growth [52]. The sensitivity of the method is quite high and even a small amount of residual biocide in a model system can be detected. Thus, to prove that the antimicrobial properties of the plastic samples under study were only manifested by contact with the surface, excluding the slow release of biocidal compounds, the PC+ plastic sample was examined in a zone-of-inhibition test. In this experiment, we used cultures of the bacteria *P. aeruginosa* and *S. aureus*. First, the microbial suspension was spread on a sterile agar plate. Then, a plastic sample (PC+), which was preliminarily immersed for 1 min in a 1% solution of compound **12a** in ethanol, was placed in the center of the Petri dish with agar. During the incubation period, the inoculum bacteria can only grow where is no antimicrobial agent. If the active agent is washed out of the sample into the environment (in this case, agar), bacterial growth in the area around the sample is suppressed. The width of this area is determined by the diffusion of the active compound in the vicinity. As can be seen in Figure 5, testing the sample on both bacterial cultures displayed no zones of inhibition, indicating no evidence of the biocidal release of **12a** from the PC+ surface. This suggests that the antimicrobial action of the tested plastic materials is only manifested by contact with the surface. From the results obtained, it is clear that a relatively stable surface coating was achieved using a simple experimental procedure.

## 3. Materials and Methods

### 3.1. The Synthesis of QPSs

QPSs based on tri-*tert*-butylphosphine were synthesized according to a previously published procedure [43].

### 3.2. Antimicrobial Activity

The antimicrobial activity of the QPSs was determined using the serial dilution technique in Mueller–Hinton Broth 2. The cultures used for testing included the Gram-positive bacteria *Staphylococcus aureus ATCC 6538P FDA 209P*, *Enterococcus faecalis ATCC 2921* and *Bacillus cereus ATCC 10702 NCTC 8035*, the Gram-negative bacteria *Escherichia coli ATCC 25922* and *Pseudomonas aeruginosa* ATCC 9027, and the fungi *Trichophyton mentagrophytes* var. gypseum 1773 and *Candida albicans ATCC 10231*; methicillin-resistant strains of *S. aureus MRSA* were obtained from hospital patients with chronic tonsillitis (*MRSA-1*) and sinusitis (*MRSA-2*) in the Republican Clinical Hospital (Kazan, Russia). Ciprofloxacin and ketoconazole were purchased from Sigma-Aldrich. The bacterial load was 3.0 × 10^5^ cfu/mL. The results were recorded every 24 h for 5 days. The cultures were incubated at 37 °C. The experiment was repeated three times. The dilutions of the compounds were prepared immediately in nutrient media; 5% aqueous solution of DMSO was used to improve the solubility of the QPSs, and the test strains were not inhibited at this concentration. The minimum inhibitory concentration (MIC) was defined as the minimum concentration of a compound that inhibits the growth of the corresponding test micro-organism. The growth of bacteria as well as the absence of growth due to the bacteriostatic action of a compound was recorded. To determine the minimal bactericidal concentration (MBC), an aliquot of the bacterial culture was transferred onto Mueller–Hinton agar in a 10 cm Petri dish and incubated for 24 h at 37 °C. The MBC was the minimal concentration at which bacterial colonies were not detected, indicating that the bacteria had been killed with an efficiency >99.9% [53].

### 3.3. Hemolytic Activity

The hemolytic activity of the QPSs was estimated by comparing the optical density of a solution containing the test compound with that of blood at 100% hemolysis. The experiments were carried out as previously described [54].

### 3.4. Cytotoxicity Assay

The cytotoxic effects of the test compounds on normal human cells were estimated by means of the multifunctional Cytell Cell Imaging system (GE Health Care Life Science, Upsala, Sweden) using the Cell Viability Bio App, which precisely counts the number of cells and evaluates their viability from fluorescence intensity based on cell staining [54]. Two fluorescent dyes that selectively penetrate the cell membranes and fluoresce at different wavelengths were used in the experiments. DAPI is able to penetrate the intact membranes of living cells and it colors the nuclei blue, while the propidium iodide dye only penetrates dead cells with damaged membranes and stains them yellow. DAPI and propidium iodide were purchased from Sigma-Aldrich. The IC_50_ was calculated using an online tool: MLA—“Quest Graph™ IC_50_ Calculator” [55]. The Chang liver cell line (human liver cells) from the N.F. Gamaleya Research Center of Epidemiology and Microbiology was used in the experiments. The cells were cultured in a standard Eagle’s nutrient medium manufactured at the Chumakov Institute of Poliomyelitis and Virus Encephalitis (PanEco company, Moscow, Russia) and supplemented with 10% fetal calf serum and 1% nonessential amino acids. The cells were plated into a 96-well plate (Eppendorf) at a concentration of 1 × 10^5^ cells/mL, with 150 μL of medium per well, and cultured in a CO_2_ incubator at 37 °C. Twenty-four hours after seeding the cells into wells, the compound under study was added at a preset dilution, at 150 μL in each well. The dilutions of the compounds were immediately prepared in nutrient medium and a 5% aqueous solution of DMSO, which is a concentration that does not induce the inhibition of cells, was added to improve the solubility of the QPSs. The experiments were repeated three times. Intact cells cultured in parallel with the experimental cells were used as a control.

### 3.5. Crystal Violet Assay

The uptake of crystal violet (CV) dye by *Staphylococcus aureus* cells was determined according to a previously published method [54]. The night culture of *S. aureus 209 P* was cultured in Mueller–Hinton broth to the middle of the exponential growth phase, centrifuged at 5000 rpm for 10 min and washed twice with 0.01 M phosphate buffer solution (PBS). The cells were resuspended in 0.01 M PBS to obtain 2 × 10^8^ cfu/mL. The inoculum was added to the test solutions of compounds in a 1:1 ratio and incubated for 30 min at 37 °C. Then, the CV dye was added to this solution to the concentration of 0.001% in the sample. Samples were incubated for 10 min, then centrifuged at 12,000 rpm for 2 min. The optical density of the supernatant was determined at a wavelength 540 nm using an Invitrologic microplate reader (Medical Biological Union, LLC, Novosibirsk, Russia). The percentage of CV uptake was calculated using the formula:

$$\mathrm{CV}\;\mathrm{uptake}\;\left(\%\right)=\frac{OD_{sample}-OD_{control}}{OD_{CV}}\times 100\%$$

### 3.6. Evaluation of the Bactericidal Properties of a Plastic Sample

The plastic samples, which were 10 mm × 10 mm × 2 mm plates, were sterilized in a medical autoclave, HG-50 (Hirayama, Japan), at 121 °C for 20 min. Then, each sample was immersed in an ethanolic solution of compound **12a** at concentrations of 0.01 and 0.001% for 1 min and air dried. After treatment, the plastic samples were placed in 24-well plates with 1 sample/well. Then, 1 mL of *S. aureus 209 P* suspension (10^5^ bacteria/mL) was added to each well. The plastic control samples were not treated with compound **12a**. The culture of *Staphylococcus aureus 209 P* was grown for a day at 37 °C in Mueller–Hinton Broth 2. Active daily growing cultures were diluted in physiological saline (NaCl 9.0 g/L) to a concentration of 10^5^ bacteria/mL and then distributed into 25-well plates containing 1 cm^2^ of coating/well, with the exception of an uncoated control well. After a 24-h incubation period at 37 °C, dilution series were performed for each treatment, and 100 μL volumes of 3 dilutions were plated on sterile Mueller–Hinton agar. After 24 h, the number of colonies on each plate was counted to obtain the corresponding concentration of live bacteria.

### 3.7. Zone of Inhibition Test

Polycarbonate plastic sample (PC+) coated with compound **12a**, which showed high antimicrobial activity in previous experiment, was chosen as a test object. This sample was tested for possible release of the active compound. Tests were performed on a culture of Gram-negative bacteria *P. aeruginosa* and Gram-positive bacteria *S. aureus*. Bacteria were plated on Petri dishes with Mueller–Hinton agar. The PC+ samples (10 mm × 10 mm × 2 mm) were immersed for 1 min in a 1% solution of **12a** in ethanol and placed on the surface of the agar plate. Then, Petri dishes were placed in an incubator at 37 °C for 24 h, and the presence of inhibition zones was evaluated.

## 4. Conclusions

The antibacterial and fungicidal activity of a broad range of sterically hindered QPSs was studied, and structure–property relationships were established. The QPSs proved to be antimicrobially active, comparably to the well-known antibiotic ciprofloxacin and the fungicidal agent ketoconazole, including against resistant strains. The phosphonium compounds with 14–16 methylene fragments in the alkyl chain were the most effective in inhibiting the growth of Gram-negative bacteria. QPSs containing 15–18 carbon atoms in the side chain demonstrated elevated activity against fungi. The bactericidal activity coupled with the fungicidal activity of some members of the QPSs under investigation could be of benefit.

The hemo- and cytotoxicity tests showed that tri-*tert*-butyl(*n*-dodecyl)phosphonium **12a** and tri-*tert*-butyl(*n*-tridecyl)phosphonium **13a** bromides exhibited low toxicity to the normal human cell lines while still showing high antibacterial activity. Thus, these compounds are safe for human health and exhibit high selectivity for pathogenic micro-organisms; consequently, they could be considered prospective drug candidates.

Surprisingly, a test for the membranotropic mechanism of action showed that QPSs have little influence on the cytoplasmic membranes of bacteria. The mechanisms of the effects of quaternary ammonium and quaternary phosphonium salts on bacteria probably differ from each other, and the influence of QPSs requires additional research. A possible mechanism of action for sterically hindered QPSs is the disruption of the balance of energy exchange inside bacterial cells.

The lead phosphonium compound **12a** was tested as an antiseptic coating for FDM polymers. It was shown to be the most effective against *S. aureus 209 P* when used to coat ABS, PLA and PETG polymers. Unexpectedly, a relatively stable surface coating was achieved using a simple application procedure. It was also shown that the antimicrobial mechanism of coated plastic samples was surface contact.

QPSs based on tri-*tert*-butylphosphine were confirmed as compounds whose structural tuning allowed the obtention of biocides that exhibited high combined activity against bacteria and fungi and possessed low toxic side effects. The further systematic study of sterically hindered quaternary phosphonium salts is therefore an area of great interest for their potential application as tunable biocides.

## Figures and Tables

**Table 1:** The structure of the QPSs under investigation.

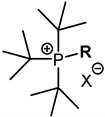		
R	X = I, Br	X = BF_4_
CH_3_	1a *	**1b**
C_2_H_5_	2a *	**2b**
*n*-C_3_H_7_	3a *	**3b**
*n*-C_4_H_9_	**4a**	**4b**
*n*-C_5_H_11_	**5a**	**5b**
*n*-C_6_H_13_	**6a**	**6b**
*n*-C_7_H_15_	**7a**	**7b**
*n*-C_8_H_17_	**8a**	**8b**
*n*-C_9_H_19_	**9a**	**9b**
*n*-C_10_H_21_	**10a**	**10b**
*n*-C_11_H_23_	**11a**	**11b**
*n*-C_12_H_25_	**12a**	**12b**
*n*-C_13_H_27_	**13a**	**13b**
*n*-C_14_H_29_	**14a**	**14b**
*n*-C_15_H_31_	**15a**	**15b**
*n*-C_16_H_33_	**16a**	**16b**
*n*-C_17_H_35_	**17a**	**17b**
*n*-C_18_H_37_	**18a**	**18b**
*n*-C_20_H_41_	**20a**	**20b**

* X = I for **1a–3a** and X = Br for **4a–18a**, **20a**.

**Table 2:** (a) Antimicrobial and fungicidal activity of QPSs: minimum inhibitory concentrations. (b) Antimicrobial and fungicidal activity of QPSs: minimum bactericidal and fungicidal concentrations.

(a)									
**Compound**	**Minimum Inhibitory Concentration (MIC), µg/mL**								
	***Sa***	***Bc***	***Ef***	***MRSA-1***	***MRSA-2***	***Ec***	***Pa***	***Tm***	***Ca***
**10a**	1.9 ± 0.1	3.9 ± 0.2	7.8 ± 0.6	1.9 ± 0.1	3.9 ± 0.2	31.3 ± 2.7	-	125 ± 11	31.3 ± 2.6
**10b**	3.9 ± 0.2	7.8 ± 0.6	15.6 ± 1.3	1.9 ± 0.1	3.9 ± 0.2	62.5 ± 5.4	-	125 ± 10	31.3 ± 2.4
**11a**	0.9 ± 0.07	1.9 ± 0.1	7.8 ± 0.5	1.9 ± 0.1	0.9 ± 0.08	31.3 ± 2.6	-	31.3 ± 2.5	62.5 ± 5.5
**11b**	0.9 ± 0.07	1.9 ± 0.1	7.8 ± 0.6	0.3 ± 0.02	0.5 ± 0.04	62.5 ± 5.3	-	125 ± 9	31.3 ± 2.4
**12a**	0.5 ± 0.03	1.9 ± 0.01	0.9 ± 0.06	0.5 ± 0.03	0.5 ± 0.03	7.8 ± 0.6	62.5 ± 5.5	15.6 ± 1.2	15.6 ± 1.4
**12b**	0.5 ± 0.03	1.9 ± 0.01	1.9 ± 0.02	1.9 ± 0.1	0.5 ± 0.04	15.6 ± 1.3	125 ± 11	31.3 ± 2.4	15.6 ± 1.3
**13a**	0.2 ± 0.01	0.9 ± 0.07	3.9 ± 0.3	0.5 ± 0.04	0.3 ± 0.02	3.9 ± 0.02	15.6 ± 1.3	125 ± 10	15.6 ± 1.2
**13b**	0.2 ± 0.01	0.5 ± 0.04	1.9 ± 0.1	0.3 ± 0.02	0.3 ± 0.01	7.8 ± 0.6	31.3 ± 2.3	62.5 ± 5.2	7.8 ± 0.5
**14a**	1.9 ± 0.2	7.8 ± 0.6	1.9 ± 0.1	0.5 ± 0.03	0.5 ± 0.04	1.9 ± 0.1	15.6 ± 1.1	125 ± 11	7.8 ± 0.6
**14b**	0.1 ± 0.008	0.9 ± 0.07	0.9 ± 0.08	0.3 ± 0.01	0.3 ± 0.02	1.9 ± 0.1	7.8 ± 0.6	15.6 ± 1.3	3.9 ± 0.3
**15a**	0.2 ± 0.01	0.5 ± 0.03	0.5 ± 0.03	0.5 ± 0.02	0.5 ± 0.04	1.9 ± 0.1	7.8 ± 0.7	62.5 ± 5.3	1.9 ± 0.1
**15b**	0.2 ± 0.01	0.5 ± 0.03	0.5 ± 0.04	0.3 ± 0.01	0.5 ± 0.03	3.9 ± 0.2	3.9 ± 0.2	62.5 ± 5.5	1.9 ± 0.1
**16a**	0.5 ± 0.04	1.9 ± 0.1	0.5 ± 0.03	1.9 ± 0.1	0.5 ± 0.03	0.9 ± 0.07	31.3 ± 2.7	-	1.9 ± 0.1
**16b**	0.5 ± 0.03	1.9 ± 0.1	0.5 ± 0.04	1.9 ± 0.1	0.5 ± 0.03	1.9 ± 0.1	3.9 ± 0.02	62.5 ± 5.6	3.9 ± 0.2
**17a**	0.2 ± 0.01	0.9 ± 0.07	1.9 ± 0.1	0.9 ± 0.06	0.9 ± 0.07	15.6 ± 1.2	7.8 ± 0.5	125 ± 10	1.9 ± 0.1
**17b**	0.5 ± 0.04	1.9 ± 0.1	0.5 ± 0.03	1.9 ± 0.1	1.9 ± 0.1	15.6 ± 1.2	7.8 ± 0.6	125 ± 10	1.9 ± 0.1
**18a**	0.9 ± 0.08	7.8 ± 0.6	0.9 ± 0.07	0.9 ± 0.07	0.9 ± 0.07	15.6 ± 1.3	15.6 ± 1.2	125 ± 11	1.9 ± 0.1
**18b**	1.9 ± 0.1	31.3 ± 2.2	1.9 ± 0.1	7.8 ± 0.6	3.9 ± 0.2	15.6 ± 1.3	15.6 ± 1.3	250 ± 19	7.8 ± 0.7
**20a**	3.9 ± 0.2	15.6 ± 1.3	3.9 ± 0.2	7.8 ± 0.6	1.9 ± 0.1	62.5 ± 5.5	31.3 ± 2.4	31.3 ± 2.3	31.3 ± 2.5
**20b**	3.9 ± 0.2	62.5 ± 5.5	7.8 ± 0.6	15.6 ± 1.3	7.8 ± 0.6	62.5 ± 5.7	125 ± 11	250 ± 18	15.6 ± 1.3
Ciprofloxacin	0.5 ± 0.03	0.5 ± 0.04	3.9 ± 0.3	125 ± 11	0.9 ± 0.07	0.5 ± 0.03	0.5 ± 0.03		
Ketoconazole								3.9 ± 0.2	3.9 ± 0.3
(b)									
**Compound**	**Minimum Bactericidal and Fungicidal Concentration (MBC, MFC), µg/mL**								
	***Sa***	***Bc***	***Ef***	***MRSA-1***	***MRSA-2***	***Ec***	***Pa***	***Tm***	***Ca***
**10a**	1.9 ± 0.1	31.3 ± 2.5	7.8 ± 0.6	15.6 ± 1.2	3.9 ± 0.2	31.3 ± 2.6	-	125 ± 11	31.3 ± 2.3
**10b**	3.9 ± 0.2	125 ± 9	15.6 ± 1.2	15.6 ± 1.3	7.8 ± 0.6	62.5 ± 5.5	-	125 ± 10	31.3 ± 2.2
**11a**	1.9 ± 0.1	125 ± 10	7.8 ± 0.6	1.9 ± 0.1	7.8 ± 0.6	31.3 ± 2.3	-	31.3 ± 2.5	62.5 ± 5.7
**11b**	3.9 ± 0.2	125 ± 10	15.6 ± 1.2	0.5 ± 0.03	0.9 ± 0.07	62.5 ± 5.4	-	125 ± 9	31.3 ± 2.1
**12a**	0.5 ± 0.03	62.5 ± 5.3	1.9 ± 0.1	0.5 ± 0.04	7.8 ± 0.6	7.8 ± 0.7	125 ± 11	15.6 ± 1.3	31.3 ± 2.7
**12b**	0.5 ± 0.03	62.5 ± 5.5	15.6 ± 1.3	0.5 ± 0.04	31.3 ± 2.7	15.6 ± 1.2	125 ± 10	62.5 ± 5.2	15.6 ± 1.3
**13a**	0.5 ± 0.04	7.8 ± 0.6	7.8 ± 0.6	0.3 ± 0.02	0.5 ± 0.03	3.9 ± 0.2	31.3 ± 2.4	125 ± 10	31.3 ± 2.2
**13b**	0.2 ± 0.01	31.3 ± 2.3	3.9 ± 0.3	0.5 ± 0.03	3.9 ± 0.3	7.8 ± 0.6	31.3 ± 2.2	62.5 ± 5.3	7.8 ± 0.6
**14a**	1.9 ± 0.1	15.6 ± 1.3	3.9 ± 0.2	7.8 ± 0.6	62.5 ± 5.3	1.9 ± 0.1	125 ± 11	125 ± 10	15.6 ± 1.2
**14b**	1.9 ± 0.1	15.6 ± 1.2	1.9 ± 0.1	0.5 ± 0.03	0.5 ± 0.03	3.9 ± 0.2	62.5 ± 5.4	15.6 ± 1.3	15.6 ± 1.2
**15a**	0.5 ± 0.03	15.6 ± 1.3	1.9 ± 0.1	0.5 ± 0.04	0.5 ± 0.03	3.9 ± 0.2	7.8 ± 0.6	62.5 ± 5.7	3.9 ± 0.2
**15b**	0.5 ± 0.03	15.6 ± 1.3	3.9 ± 0.2	0.3 ± 0.01	0.5 ± 0.04	3.9 ± 0.2	3.9 ± 0.3	62.5 ± 5.2	15.6 ± 1.2
**16a**	0.5 ± 0.03	31.3 ± 2.2	0.5 ± 0.03	7.8 ± 0.6	7.8 ± 0.7	1.9 ± 0.1	125 ± 9	-	3.9 ± 0.3
**16b**	0.5 ± 0.03	31.3 ± 2.4	0.5 ± 0.03	15.6 ± 1.2	3.9 ± 0.2	1.9 ± 0.1	62.5 ± 5.3	62.5 ± 5.5	3.9 ± 0.2
**17a**	0.5 ± 0.03	15.6 ± 1.2	1.9 ± 0.1	0.9 ± 0.07	0.9 ± 0.06	15.6 ± 1.3	7.8 ± 0.7	125 ± 10	3.9 ± 0.2
**17b**	0.5 ± 0.04	15.6 ± 1.3	3.9 ± 0.2	1.9 ± 0.1	1.9 ± 0.1	7.8 ± 0.6	15.6 ± 1.3	125 ± 11	7.8 ± 0.7
**18a**	0.9 ± 0.07	31.3 ± 2.5	0.9 ± 0.07	15.6 ± 1.2	0.9 ± 0.07	15.6 ± 1.1	62.5 ± 5.6	125 ± 9	3.9 ± 0.2
**18b**	3.9 ± 0.2	125 ± 11	3.9 ± 0.2	15.6 ± 1.2	3.9 ± 0.3	62.5 ± 5.4	31.3 ± 2.5	250 ± 19	15.6 ± 1.2
**20a**	3.9 ± 0.2	31.3 ± 2.6	7.8 ± 0.6	31.3 ± 2.7	15.6 ± 1.2	62.5 ± 5.5	125 ± 11	31.3 ± 2.5	62.5 ± 5.7
**20b**	3.9 ± 0.3	-	15.6 ± 1.3	15.6 ± 1.2	7.8 ± 0.7	62.5 ± 5.5	-	250 ± 18	31.3 ± 2.1
Ciprofloxacin	0.5 ± 0.03	0.5 ± 0.04	3.9 ± 0.3	250 ± 19	0.9 ± 0.06	0.5 ± 0.03	0.5 ± 0.03		
Ketoconazole								3.9 ± 0.2	3.9 ± 0.3

Average of three values measured; ± standard deviation (SD); - means non-active.

**Table 3:** Polymers for 3D printing.

FDM Polymers	Abbreviation
acrylonitrile butadiene styrene	ABS
polylactic acid	PLA natural
polylactic acid modified	PLA+
polyethylene terephthalate glycol	PETG
polycarbonate modified	PC+
nylon	Nylon

**Table 4:** Evaluation of bactericidal properties of a plastic sample (1 cm^2^) after 24 h of incubation with 10^5^ *S. aureus 209 P*.

Concentration of 12a, %	CFU/cm^2^					
	Name of Plastic Samples					
	ABS	PLA Natural	PLA+	PETG	PC+	Nylon
0 ^a^	1.3 × 10^4^	4.0 × 10^4^	8.0 × 10^4^	5.0 × 10^4^	3.6 × 10^4^	4.5 × 10^4^
0.01	-	-	-	-	-	2.0 × 10^2^
0.001	2.5 × 10^3^	5.0 × 10^3^	1.6 × 10^4^	1.8 × 10^3^	1.2 × 10^4^	3.2 × 10^4^

^a^ A plastic control sample without test-compound treatment; -: no growth.

## Data Availability

Not applicable.

## Supplementary Materials

The following supporting information can be downloaded at: https://www.mdpi.com/article/10.3390/ijms23010086/s1.
