# Supplementary material for: Sterically Hindered Quaternary Phosphonium Salts (QPSs): Antimicrobial Activity and Hemolytic and Cytotoxic Properties
Source: Int J Mol Sci. 2021 Dec 22;23(1):86. doi: 10.3390/ijms23010086 (PMC8744835; doi:10.3390/ijms23010086)
Supplement: Supplementary file 1 [file ijms-23-00086-s001.zip › ijms-1377656-supplementary.pdf]

Supplementary Materials

"Sterically Hindered Quaternary Phosphonium Salts (QPSs): Antimicrobial Activity and Hemolytic and Cytotoxic Properties"

By V.V. Ermolaev[1], D.M. Arkhipova[1,2]*, V.A. Miluykov[1], A.P. Lyubina[1], S.K. Amerhanova[1], N.V. Kulik[1], A.D. Voloshina[1], V.P. Ananikov[2]

[1] Arbuzov Institute of Organic and Physical Chemistry, FRC Kazan Scientific Center, Russian Academy of Sciences, Arbuzov str. 8, Kazan, 420088, Russian Federation; ermolaev@iopc.ru

[2] N.D. Zelinsky Institute of Organic Chemistry, Russian Academy of Sciences, Leninsky prospect, 47, Moscow, 119991, Russian Federation

* Correspondence: arkhipova_daria@ioc.ac.ru

Table S1. Antimicrobial activity of QPSs. Minimum inhibitory concentration.

Compound	Minimum inhibitory concentration (MIC), µg/ml								
	Sa	*Bc*	*Ef*	*MRSA-1*	*MRSA-2*	*Ec*	*Pa*	*Tm*	*Ca*
**1a**	-	-	-	-	-	-	-	-	-
**1b**	-	-	-	-	-	-	-	-	-
**2a**	-	-	-	-	-	-	-	-	-
**2b**	-	-	-	-	-	-	-	-	-
**3a**	-	-	-	-	-	-	-	-	-
**3b**	-	-	-	-	-	-	-	-	-
**4a**	-	-	-	-	-	-	-	-	-
**4b**	-	-	-	-	-	-	-	-	-
**5a**	-	-	-	-	-	-	-	-	-
**5b**	-	-	-	-	-	-	-	-	-
**6a**	125±11	-	-	-	-	-	-	-	-
**6b**	250±19	-	-	-	-	-	-	-	-
**7a**	62.5±5.5	125±10	125±	-	-	-	-	-	-
**7b**	62.5±	125±	125±	-	-	-	-	-	-
**8a**	15.6±	31.3±	62.5±	15.6±	31.3±	125±	-	-	-
**8b**	31.3±	62.5±	62.5±	31.3±	62.5±	-	-	-	-
**9a**	7.8±	15.6±	62.5±	7.8±	15.6±	125±	-	-	-
**9b**	15.6±	31.3±	31.3±	15.6±	31.3±	250±	-	-	-
Ciprofloxacin	0.5±0.03	0.5±0.04	3.9±0.3	125±11	0.9±0.07	0.5±0.03	0.5±0.03		
Ketoconazole								3.9±0.2	3.9±0.3

Average of three values measured; ± standard deviation (SD); - means non-active

Table S1 (cont.). Antimicrobial activity of QPSs. Minimum bactericidal and fungicidal concentration.

Compound	Minimum bactericidal and fungicidal concentration (MBC, MFC), µg/ml								
	Sa	*Bc*	*Ef*	*MRSA-1*	*MRSA-2*	*Ec*	*Pa*	*Tm*	*Ca*
**1a**	-	-	-	-	-	-	-	-	-
**1b**	-	-	-	-	-	-	-	-	-
**2a**	-	-	-	-	-	-	-	-	-
**2b**	-	-	-	-	-	-	-	-	-
**3a**	-	-	-	-	-	-	-	-	-
**3b**	-	-	-	-	-	-	-	-	-
**4a**	-	-	-	-	-	-	-	-	-
**4b**	-	-	-	-	-	-	-	-	-
**5a**	-	-	-	-	-	-	-	-	-
**5b**	-	-	-	-	-	-	-	-	-
**6a**	-	-	-	-	-	-	-	-	-
**6b**	-	-	-	-	-	-	-	-	-
**7a**	-	-	-	-	-	-	-	-	-
**7b**	62.5±	-	-	-	-	-	-	-	-
**8a**	62.5±	-	-	-	-	125±	-	-	-
**8b**	31.3±	-	125±	-	-	-	-	-	-
**9a**	15.6±	125±	125±	-	-	125±	-	-	-
**9b**	15.6±	-	125±	-	-	250±	-	-	-
Ciprofloxacin	0.5±0.03	0.5±0.04	3.9±0.3	250±19	0.9±0.06	0.5±0.03	0.5±0.03		
Ketoconazole								3.9±0.2	3.9±0.3

Average of three values measured; ± standard deviation (SD); - means non-active

Table S2. Hemotoxic and cytotoxic activity of QPSs, expressed in terms of HC_{50} and IC_{50}.

Compounds	HC_{50}, µg/ml	IC_{50}, µg/ml
**1a**	>125	>100
**1b**	>125	>100
**2a**	>125	>100
**2b**	>125	>100
**3a**	>125	>100
**3b**	>125	>100
**4a**	>125	>100
**4b**	>125	>100
**5a**	>125	>100
**5b**	>125	>100
**6a**	>125	>100
**6b**	>125	>100
**7a**	>125	>100
**7b**	>125	>100
**8a**	>125	>100
**8b**	>125	>100
**9a**	>125	>100
**9b**	>125	>100
**10a**	>125	66
**10b**	>125	71
